# Supplementary material for: Dynamic allostery in the peptide/MHC complex enables TCR neoantigen selectivity
Source: Nat Commun. 2025 Jan 20;16:849. doi: 10.1038/s41467-025-56004-8 (PMC11756396; doi:10.1038/s41467-025-56004-8)
Supplement: Supplementary file 1 — Supplementary Information [file 41467_2025_56004_MOESM1_ESM.pdf]

SUPPLEMENTARY INFORMATION

Dynamic allostery in the peptide/MHC complex enables TCR neoantigen selectivity

Jiaqi Ma[1,2,*], Cory M. Ayres[1,2,*], Chad A. Brambley[1,2], Smita S. Chandran[3,4], Tatiana J. Rosales[1,2], W. W. J. Gihan Perera[1,2], Bassant Eldaly[1,2], William T. Murray [3,4], Steven A. Corcelli[1], Evgenii L. Kovrigin[1], Christopher A. Klebanoff[3,4,5,6], and Brian M. Baker[1,2,**]

1. Department of Chemistry and Biochemistry, University of Notre Dame, Notre Dame, IN, USA
2. Harper Cancer Research Institute, University of Notre Dame, Notre Dame, IN, USA.
3. Human Oncology and Pathogenesis Program, Memorial Sloan Kettering Cancer Center (MSKCC), New York, NY, USA.
4. Center for Cell Engineering, MSKCC, New York, NY, USA.
5. Weill Cornell Medical College, New York, NY, USA.
6. Parker Institute for Cancer Immunotherapy, New York, NY, USA.

*Equal author contribution.

**Corresponding author: brian-baker@nd.edu

This document includes:

Supplementary Figures 1 to 13

Supplementary Tables 1 to 4

Supplementary Note 1

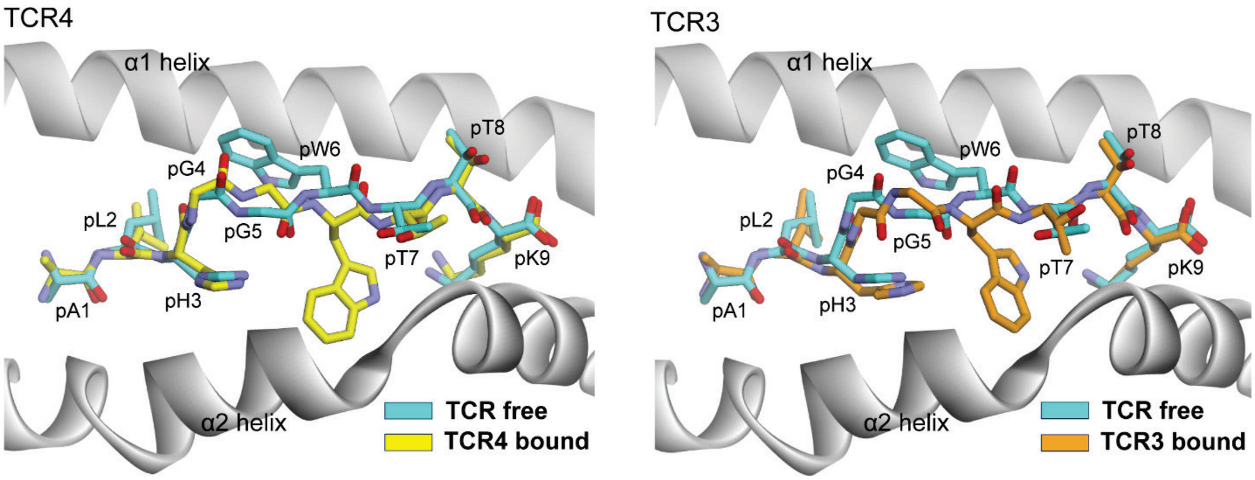

Supplementary Figure 1. Detailed views of the conformational changes in the neoantigen upon TCR binding. The change upon binding of TCR4 is in the left panel; TCR3 is in the right panel. Superimpositions are through the Cα atoms of residues 1-180 of the HLA-A3 peptide binding groove.

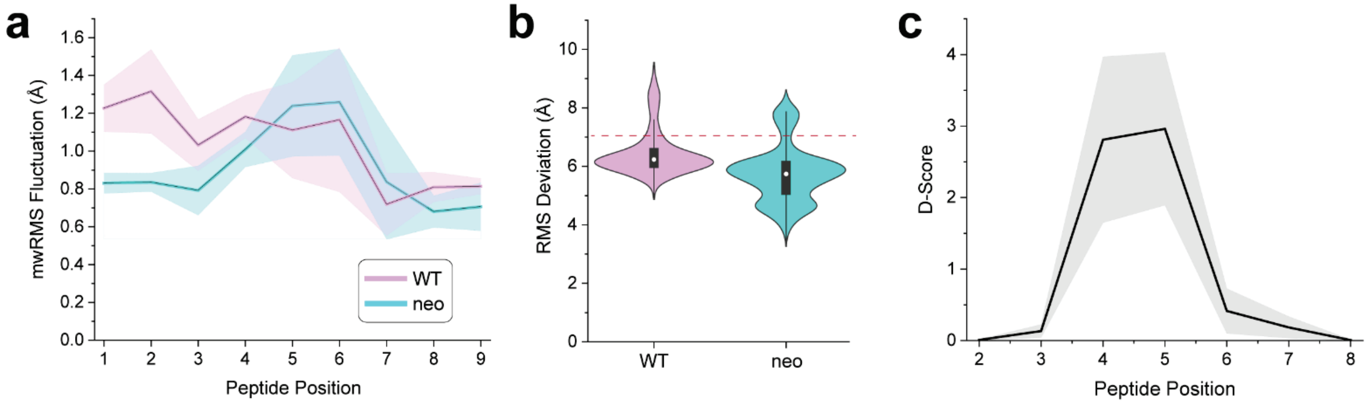

Supplementary Figure 2. Divergent motions of the WT peptide and neoantigen bound to HLA-A3 are evident across replicate molecular dynamics simulations. **A)** Mass-weighted RMS fluctuations across the peptides from four independent pairs of 2 μs peptide/HLA-A3 simulations. Solid lines give the average, shaded regions the standard deviations. Compare to Fig. 4A, which shows data from the first pairs of simulations. **B)** Distribution of RMS deviations for pTrp6 relative to its conformation in the TCR4-bound complex in the four pairs of simulations. Dashed line is the value from superimposition of TCR4-bound and free neoantigen/HLA-A3 structures. Compare to Fig. 4B, which shows time series data from the first pairs of simulations. White circles give the average; black rectangles the IQR. **C)** D-score analysis of the peptides from the four pairs of simulations. The solid line represents the average, the shaded region the standard deviation. Compare to Fig. 4C, which shows data from the first pairs of simulations.

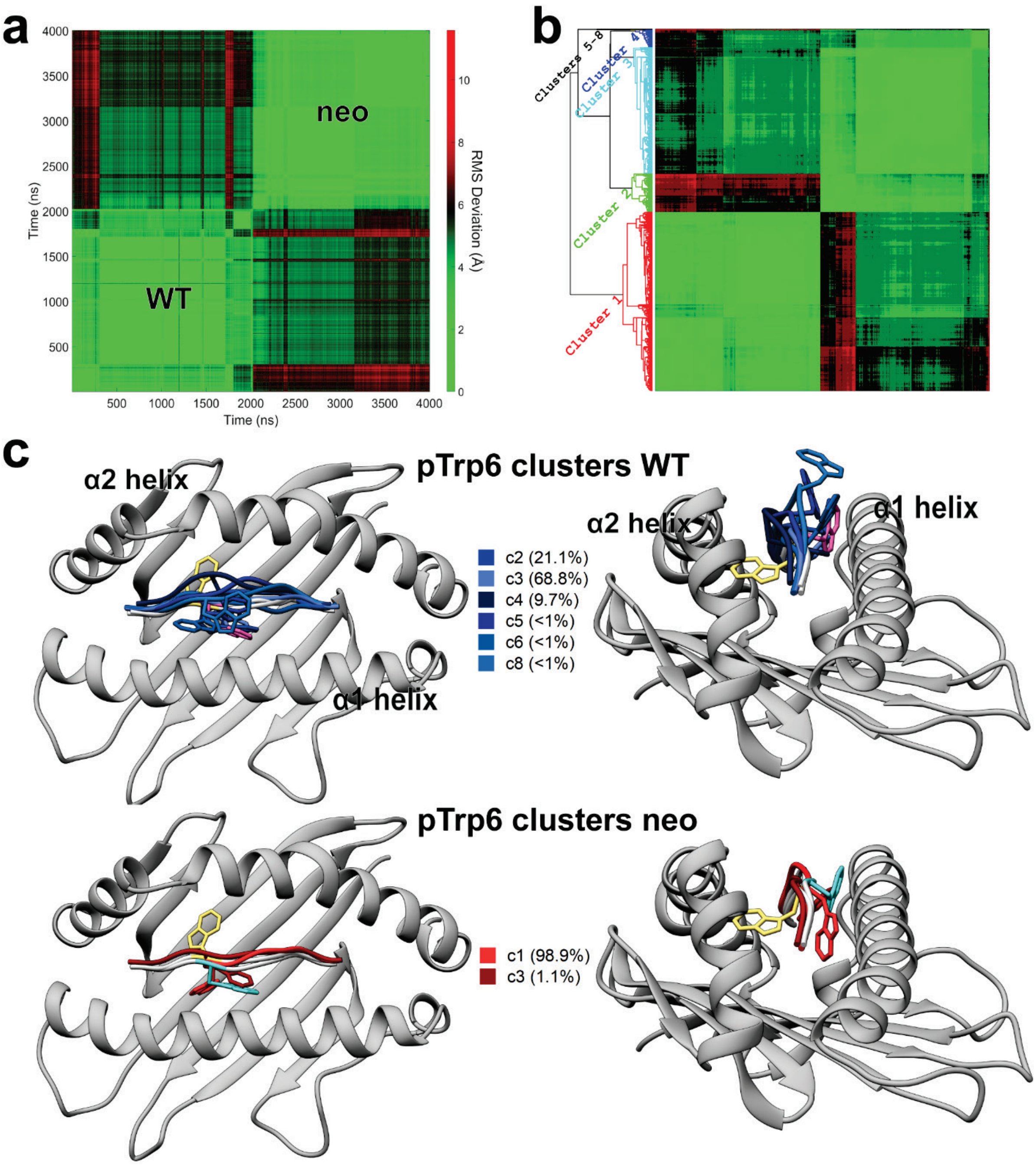

Supplementary Figure 3. Conformational clustering reveals different conformational states sampled by pTrp6 in the neoantigen and WT peptide/HLA-A3 complexes. **A)** 2D RMSD analysis of the pTrp6 amino acid over the course of the neoantigen and WT peptide/HLA-A3 simulations after superimposition of the HLA-A3 binding groove. Quadrants for the WT and neoantigen simulations are indicated; the much higher values in the cross-simulation quadrants illustrate the different conformational sampling. **B)** Cluster analysis of the 2D RMSD data from panel A (supplemented with TCR-free and TCR4-bound coordinates as indicated in the Methods). pTrp6 clustered into 8 major conformations as indicated. Cluster 2 reflects the TCR-free and Cluster 7 (not sampled during the simulations) reflects the TCR4-bound conformations. **C)** Visualization of the pTrp6 conformational clusters for the WT peptide (top) and neoantigen (bottom), showing the tendency for pTrp6 to move above the backbone in the WT simulation, but below the backbone in the neoantigen simulation. The crystallographic coordinates of the TCR-free WT, TCR-free neoantigen, and TCR4-bound neoantigen are colored magenta, cyan, and yellow, respectively.

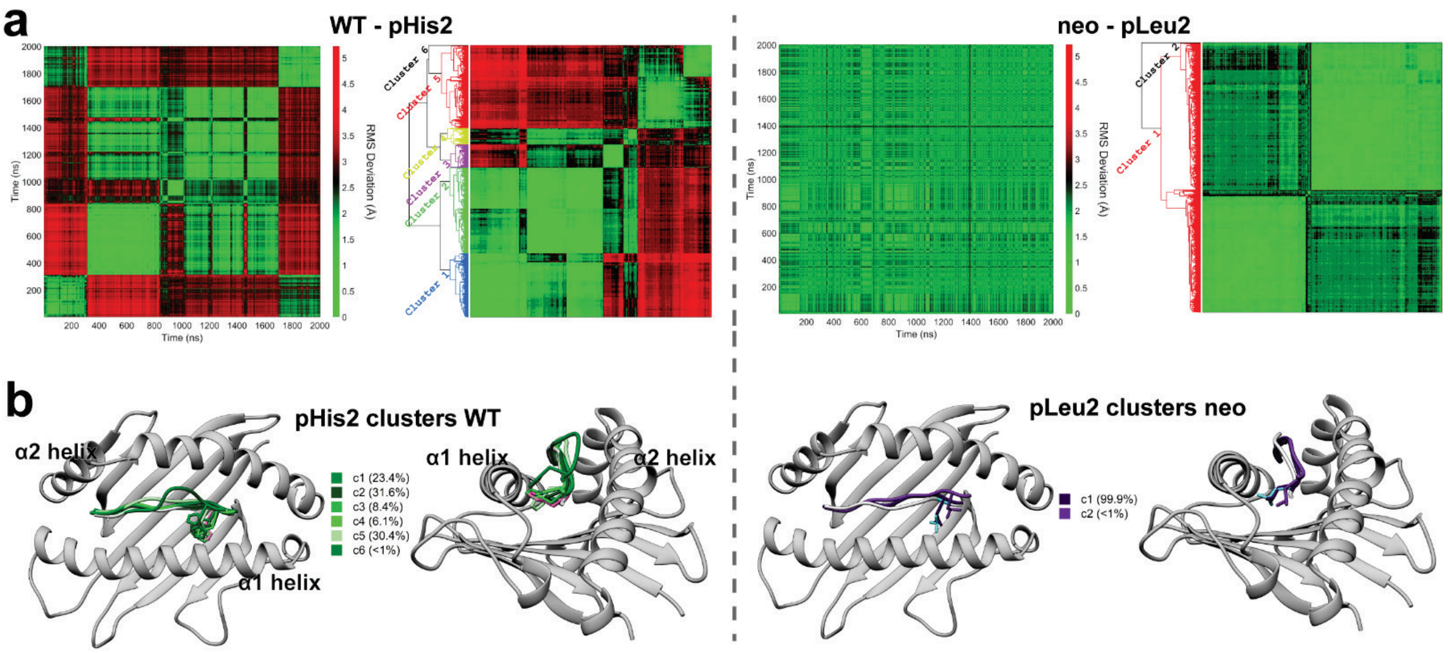

Supplementary Figure 4. Conformational clustering illustrates the significant dynamic differences between the position 2 amino acid in the neoantigen and WT peptide. **A)** 2D RMSD analysis and conformational clustering for pHis2 in the WT simulation (left) and pLeu2 in the neoantigen simulation (right), illustrating the substantially greater conformational diversity for the position 2 anchor in the WT vs. neoantigen. **B)** Visualization of the position 2 conformational clusters for the WT peptide (left) and neoantigen (right), further illustrating the more dynamic behavior of position 2 in the WT peptide. The crystallographic coordinates of the WT and TCR-free neoantigen are colored magenta and cyan, respectively.

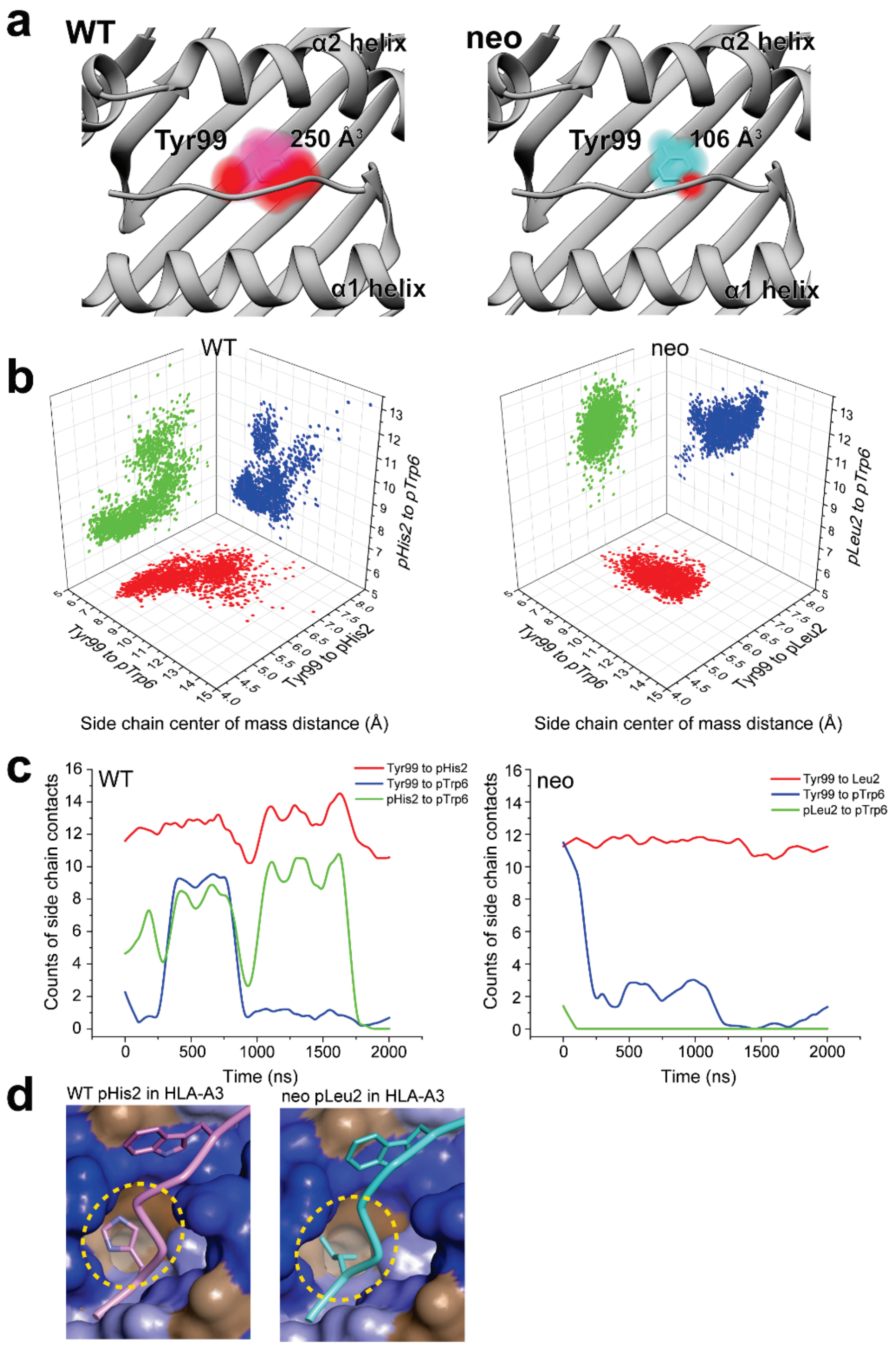

Supplementary Figure 5. Side chains move closer and form more extensive interactions with pTrp6 in the WT compared to neoantigen simulations. **A)** Conformational space occupied by the HLA-A3 Tyr99 side chain during the simulations with the WT peptide (left) and neoantigen (right). Color density reflects degree of sampling (voxels sampled <10% of the time excluded), values give volumes of sampled space. The red space corresponds to the tyrosine hydroxyl. Substantially more volume is sampled in the WT compared to the neoantigen simulation. **B)** Inter-side chain distances from the peptide/HLA-A3 simulations for pHis2/pLeu2, pTrp6, and Tyr99 of HLA-A3, measured by distances between side chain centers of mass. Points are for each ns of the 2 µs simulations. Data for the WT peptide are on the left; data for the neoantigen are on the right. The three side chains are more dynamic and move closer to each other in the WT compared to the neoantigen simulation. **C)** Counts of side chain-side chain contacts for pHis2/pLeu2, pTrp6, and Tyr99 of HLA-A3 as a function of time during the two simulations. More contacts are made in the WT simulation (left), particularly between peptide position 2 and pTrp6 and Tyr99 and pTrp6 (data were smoothed using LOWESS; see Supplementary Fig. 13 for unsmoothed data). **D)** Visualization of how pHis2 in the WT peptide and pLeu2 in the neoantigen fit into the HLA-A3 B pocket in the respective crystallographic structures. pLeu2 of the neoantigen reaches further into the pocket, whereas pHis2 lies in the depression. Surface is colored according to amino acid hydrophobicity, from blue (hydrophilic) to brown (hydrophobic).

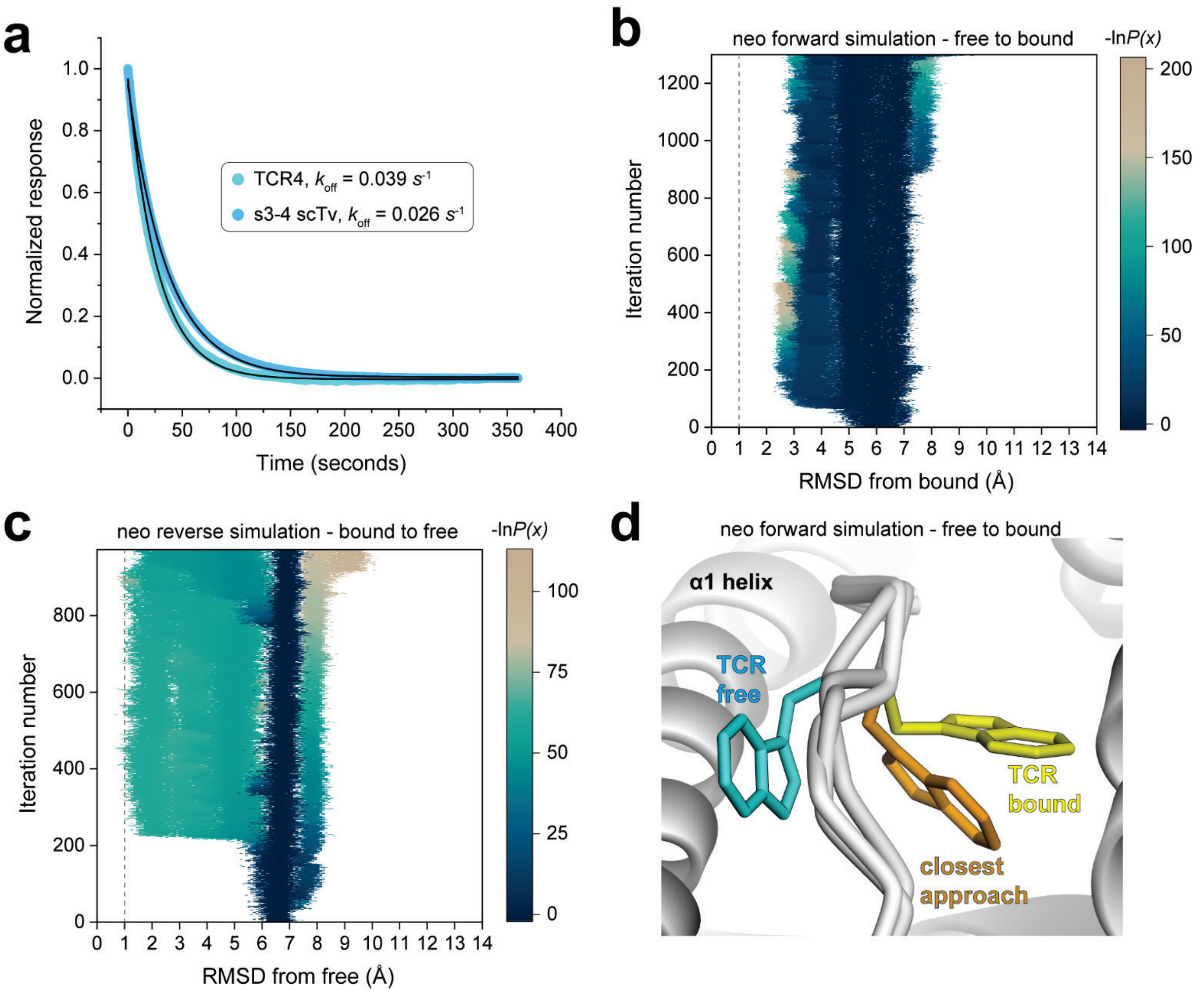

Supplementary Figure 6. Correlates with the under-peptide mechanism for the flip in the neoantigen. **A)** Dissociation phases from SPR kinetic experiments, showing the dissociation rates for TCR4 and the s3-4 scTv from the neoantigen/HLA-A3 complex. Solid lines are fits to single exponential decay functions, with the k_{off} values indicated. Together with the K_D values in Fig. 1c, the dissociation rates provide the association rates via $k_{on} = k_{off}/K_D$. **B)** Evolution of the neoantigen/HLA-A3 WEMD simulations in the forward direction, showing probability as a function of iteration number and RMSD from the target, in this case the conformation of pTrp6 in the ternary complex with TCR4 after starting from the TCR-free conformation. In 1300 WEMD iterations, pTrp6 approaches but does not reach the TCR-bound conformation. A successful transition was described as an RMSD < 1 Å, indicated by the dashed line. **C)** As in panel B, but for a reverse simulation, showing probability vs. iteration and the RMSD of pTrp6 from the TCR-free conformation after starting from the conformation in the TCR4 ternary complex. In 970 WEMD iterations, a successful transition was observed 109 times. **D)** Closest approach of pTrp6 to its TCR4 bound conformation in the forward WEMD simulations, indicating that even though a successful transition was not observed, the side chain still traversed under the peptide backbone to the adjacent side of the binding groove. RMSD to bound is 2.2 Å.

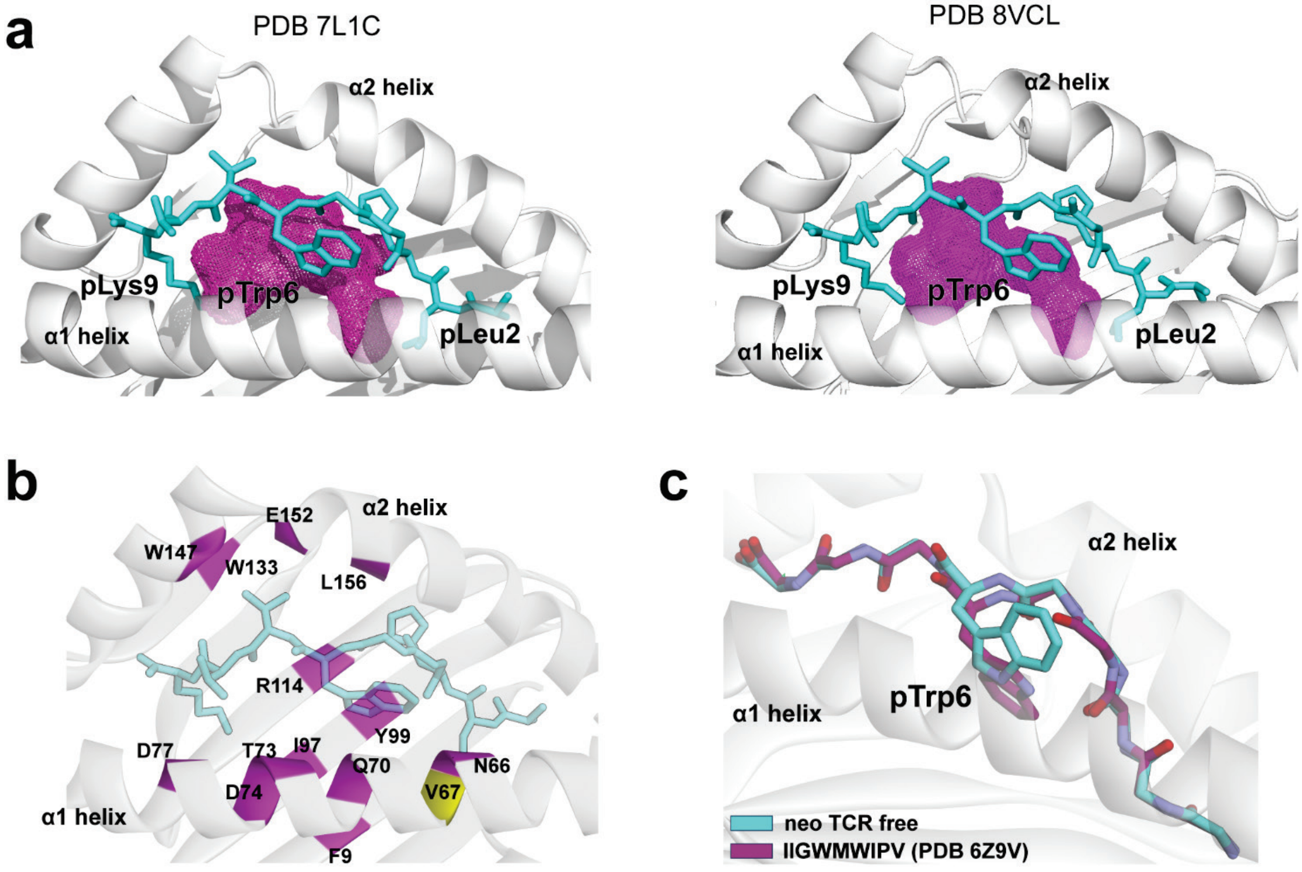

Supplementary Figure 7. Cavities between the neoantigen and the HLA-A3 peptide binding groove**. A)** The cavity under the peptide in the neoantigen/HLA-A3 complex, computed with the original structure (left: PDB 7L1C; volume of 401 Å^3) or the replicate structure determined here (right: PDB 8VCL; volume of 284 Å^3). **B)** Residues lining the cavity in the neoantigen/HLA-A3 complex. The yellow position on the α1 helix (V67) participates in the cavity in the original structure (PDB 7L1C) but not the replicate (PDB 8VCL), helping to explain the differing volumes. **C)** Example of a peptide presented by HLA-A2 with a backbone conformation almost identical to that of the PI3Kα neoantigen bound to HLA-A3 and with pTrp6 pointing down into the base of the binding groove, resembling a snapshot midway through the neoantigen transition (PDB 6Z9V).

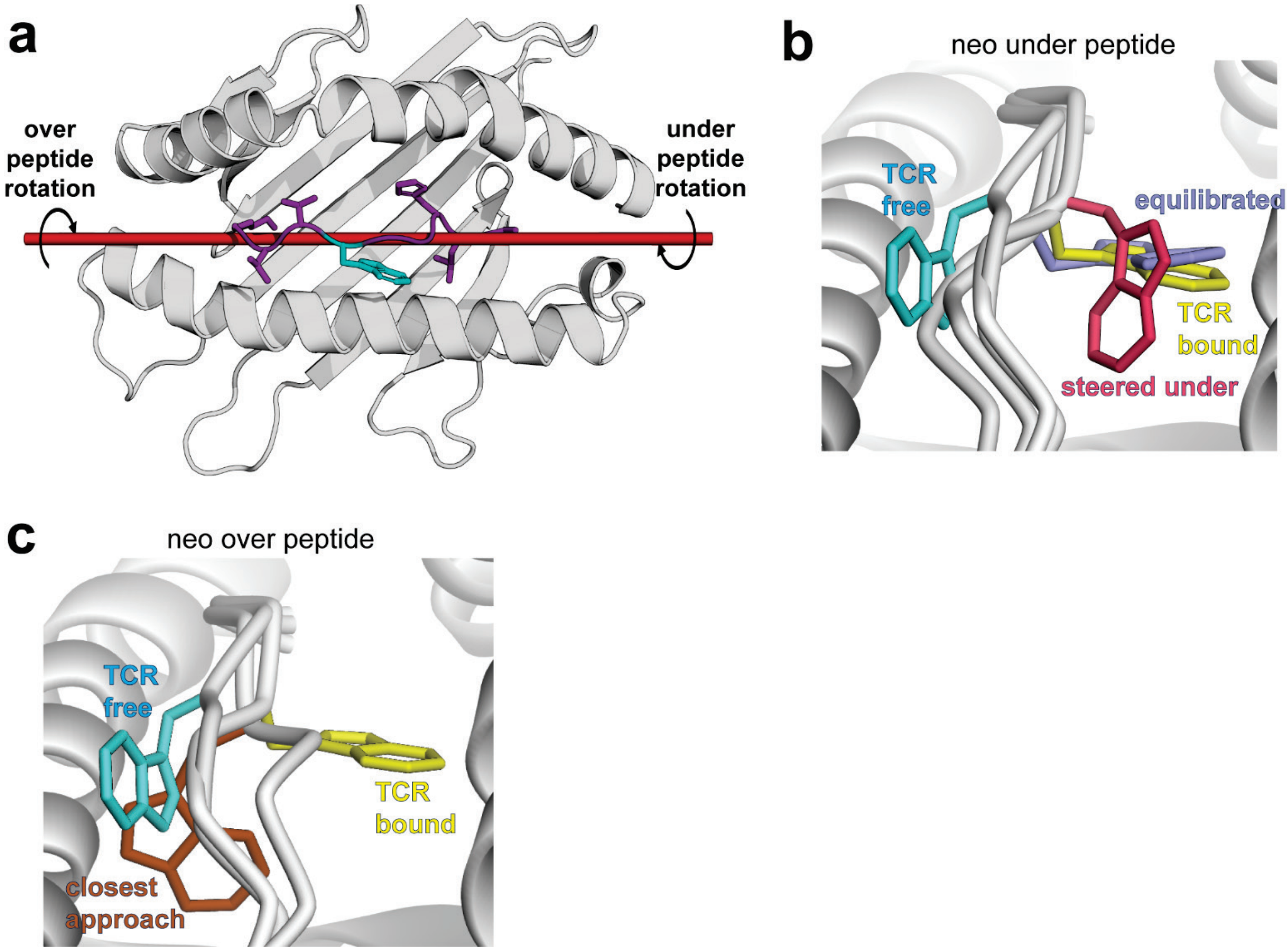

Supplementary Figure 8. Steered molecular dynamics comparing the under-peptide with the over-peptide transition. **A)** Diagram showing the axis and directions for under- and over-peptide enforced rotation performed by SMD. The pTrp6 amino acid was defined as the rotation group, with the axis of rotation set as a line through the peptide backbone as indicated. **B)** Closest approach of the pTrp6 side chain to the TCR4-bound state for the SMD under-peptide rotation (RMSD to bound is 3.7 Å, achieved with a spring constant of 100 kJ/mol/nm^2). Unrestrained traditional molecular dynamics simulations on the neoantigen/HLA-A3 complex starting from the closest approach resulted in pTrp6 adopting the TCR4-bound state within 35 ns of simulation time (final RMSD to bound is 0.7 Å; indicated as equilibrated). **C)** Closest approach of the pTrp6 side chain to the TCR4-bound state for the SMD over-peptide rotation (RMSD to bound is 5.8 Å, achieved with a spring constant of 800 kJ/mol/nm^2).

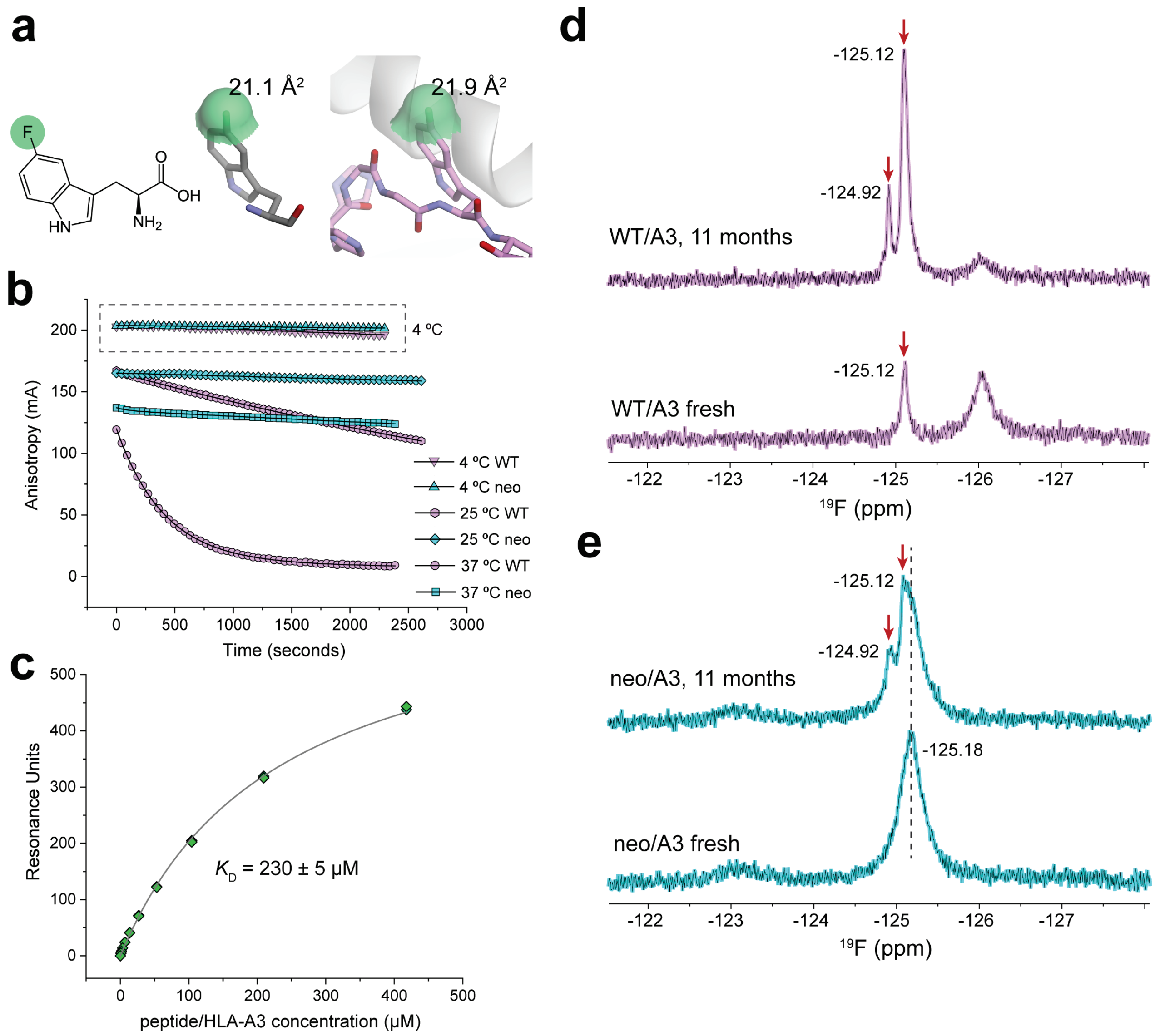

Supplementary Figure 9. Fluorine modification of pTrp6 in the neoantigen and WT peptide/HLA-A3 complexes and the impact of sample degradation over time. **A)** 5-fluoro-tryptophan and its incorporation into the peptide/HLA-A3 complexes. The fluorine is exposed when modeled in the static structures, as indicated by its solvent accessible surface in the complex with the WT peptide (green surface). **B)** Neoantigen and WT peptide dissociation kinetics from HLA-A3 as a function of temperature, measured by fluorescence anisotropy. While the more rapid dissociation of the WT peptide is evident at higher temperatures, at 4 °C the dissociation kinetics are significantly slowed (37 °C data from ref. 15). **C)** The fluorine modified neoantigen is compatible with TCR binding, as indicated by the binding of TCR3 to the 5F-Trp modified neoantigen/HLA-A3 complex. K_D value is from a fit to a single dataset with duplicate injections; error is standard error of the fit. **D)** The -125.12 ppm resonance in the WT peptide/HLA-A3 complex is from sample degradation, as indicated by comparison of a fresh sample with a sample stored at 4 °C for approximately 11 months, in which the sharp -125.12 ppm resonance is substantially increased and the other major resonances diminished. The -125.12 ppm resonance matches the position of the ^{19}F in the free WT peptide yet has a broader linewidth, indicating an exposed fluorine with residual interaction with the protein. In addition, another resonance at -124.92 ppm has emerged, reflecting further degradation of the complex. **E)** The new resonances emerging in an aged sample of the neoantigen/HLA-A3 complex. After 11 months, the peak at -125.18 has developed a shoulder at -125.12 ppm, and a peak has emerged at approximately -124.92 ppm. This indicates a degradation pathway similar to that of the WT complex, although degradation is less pronounced, consistent with the greater stability of the neoantigen/HLA-A3 complex.

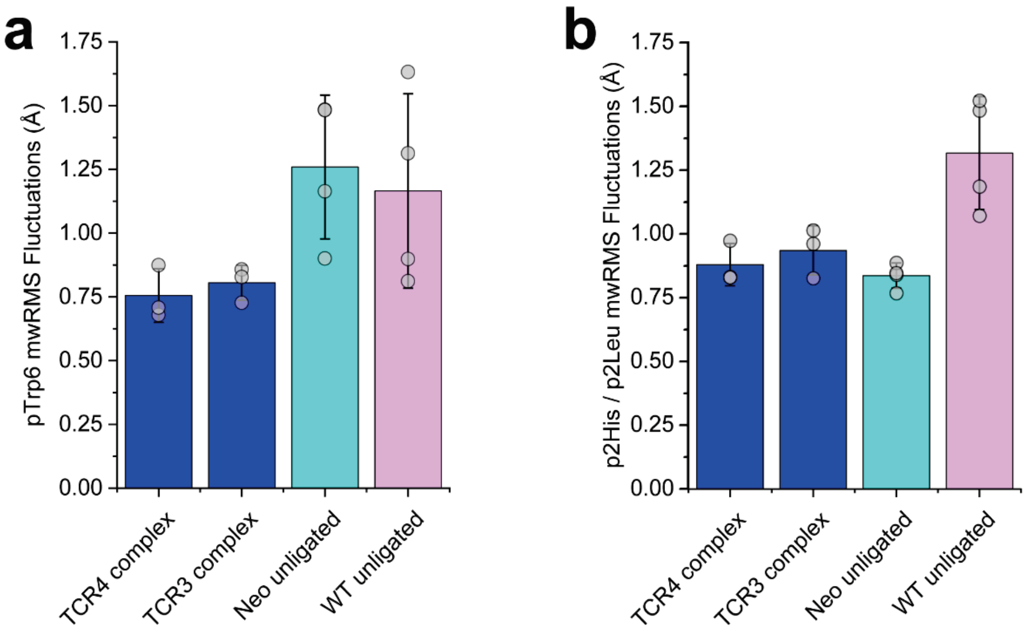

Supplementary Figure 10. The rigidity of pTrp6 in the neoantigen bound to TCR3 and TCR4 is evident across replicate molecular dynamics simulations. **A)** Data shown are the averages and standard deviations of mass weighted RMS fluctuations for pTrp6 from three independent 1 μs simulations of complexes with TCR3 and TCR4. Values shown indicate averages with standard deviations as error bars. For comparison, the fluctuations for the unligated (TCR-free) neoantigen and WT peptide/HLA-A3 complexes from four independent 2 μs simulations are also shown. **B)** Same as in panel A, but for peptide position 2 (leucine for simulations with the neoantigen; histidine for the simulation with the WT peptide). For both panels, the data from each simulation are superimposed as grey points.

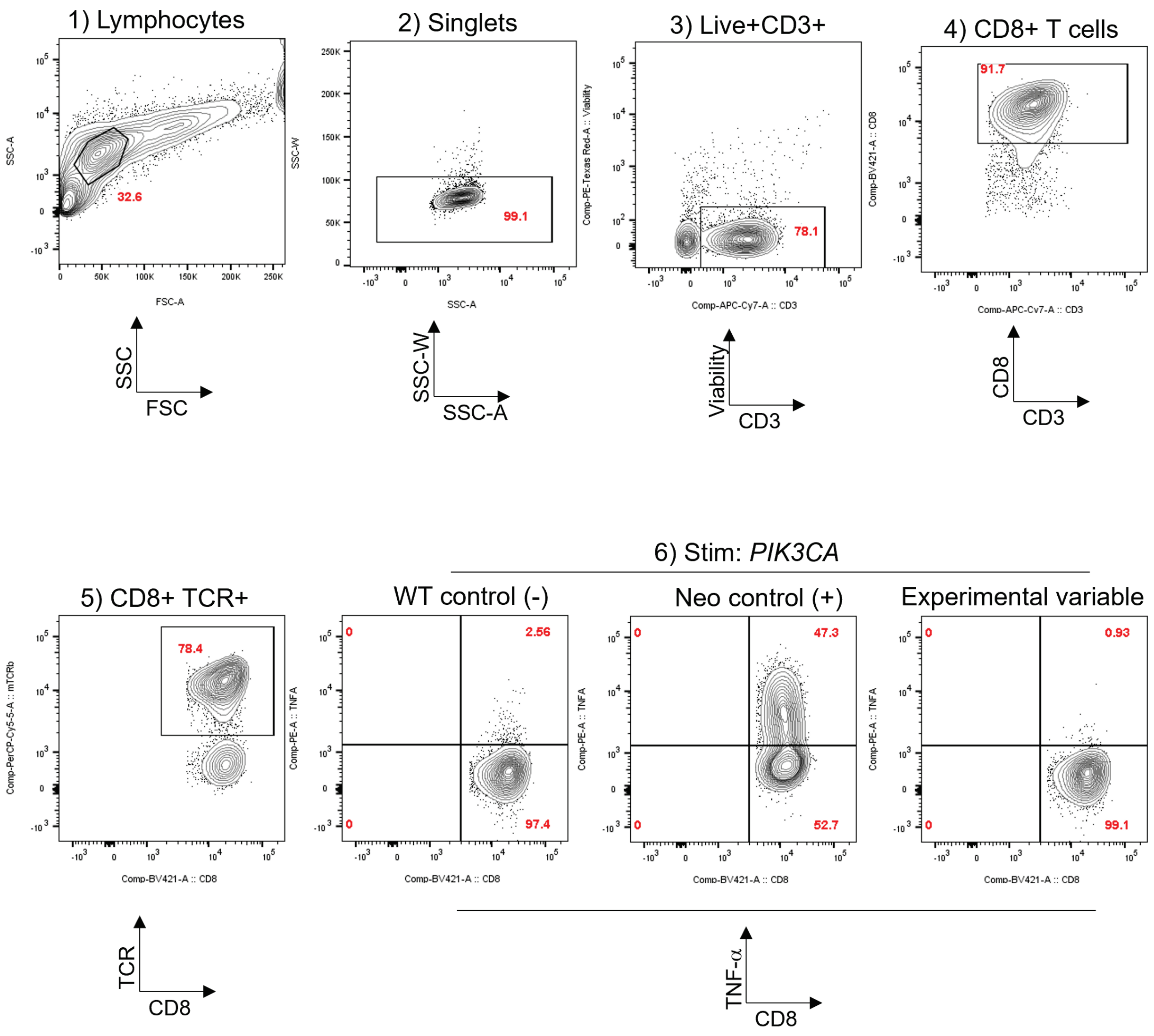

Supplementary Figure 11. Representative flow cytometry gating strategy to determine cytokine production of TCR transduced T cells.

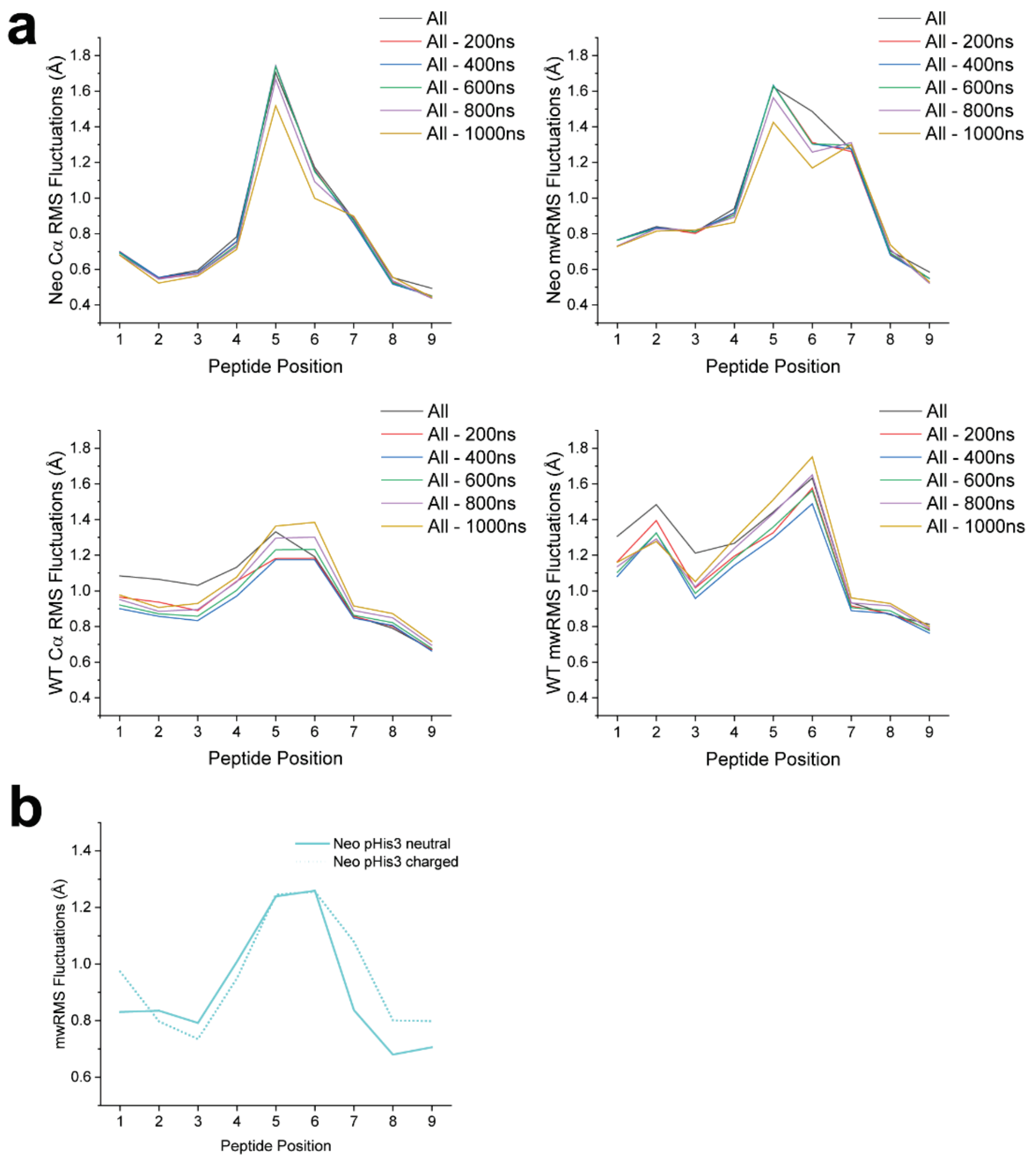

Supplementary Figure 12. **A)** Time course analysis for demonstrating equilibrated and stable MD simulations. For the 2 μs simulations of the free peptide/HLA-A3 molecules, the peptide Cα and mass weighted RMS fluctuations were calculated for both the entire trajectories, as well as for various trajectories which removed portions of the beginning of the simulation (indicated by All and All - x ns). The trends for the neoantigen and WT simulations were maintained, and differences were all less than 0.25 Å. These results indicate that our pre-production method generates equilibrated and stable systems. **B)** Comparison of mass weighted RMS fluctuations across the peptide from simulations when pHis3 of the neoantigen in the peptide/HLA-A3 complex was either neutral or charged. The behavior was the same in either case, and all simulations thus used neutral pHis3.

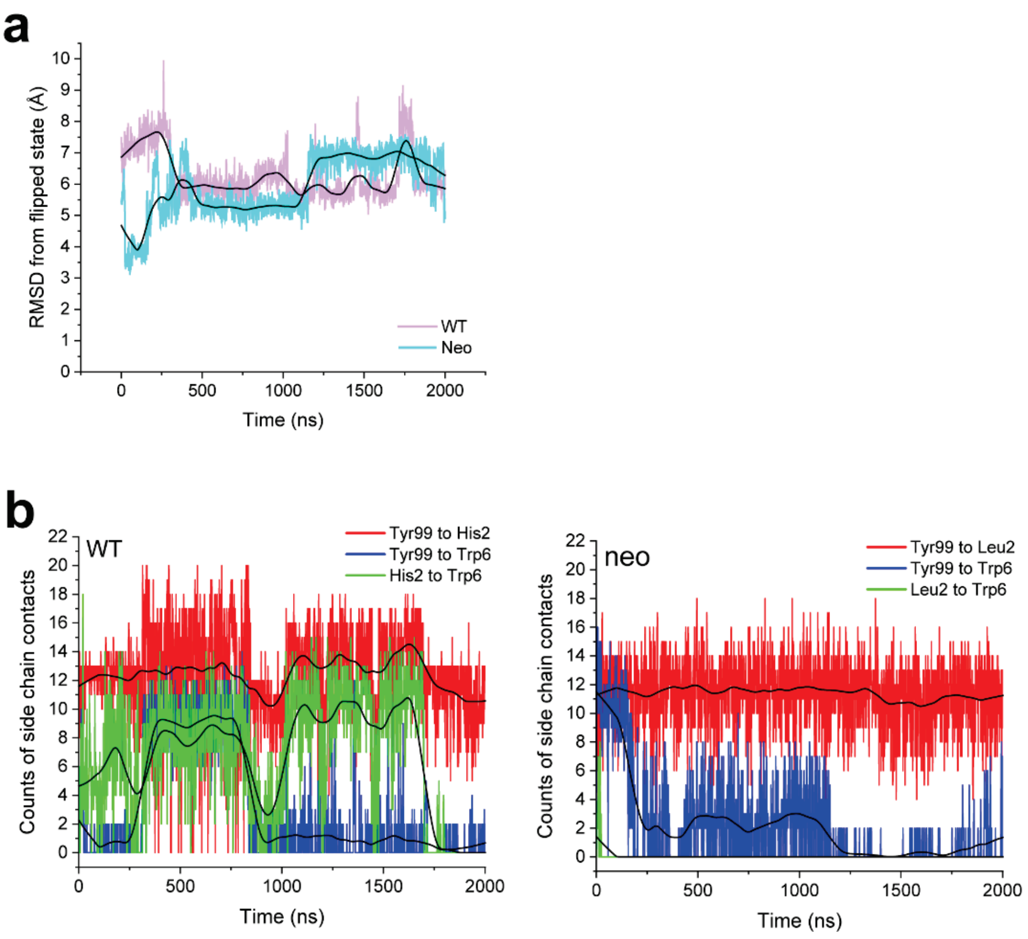

Supplementary Figure 13. Unsmoothed RMSD and interaction count data. **A)** Unsmoothed RMSD values for pTrp6 relative to its conformation in the ternary complex with TCR4, indicating that the side chain does not flip in either peptide/HLA-A3 simulation. Solid black lines show the LOWESS smoothed data shown in Fig. 4B. **B)** Unsmoothed counts of side chain/side chain contacts for pHis2/pLeu2, pTrp6, and Tyr99 of HLA-A3 as a function of time during the two simulations. More contacts are made in the WT simulation, particularly for position 2-pTrp6 and Tyr99-pTrp6. Solid black lines show the LOWESS smoothed data shown in Supplementary Fig. 5C.

Supplementary Table 1. X-ray data collection and refinement statistics for the replicate neoantigen/HLA-A3 complex and the Bta6-substituted neoantigen/HLA-A3 complex.*

	**neoantigen/HLA-A3**	**Bta6-neoantigen/HLA-A3**
PDB accession code	8VCL	9ASG
Resolution range (Å)	43.98 - 2.4 (2.486 - 2.4)	44.02 - 2.03 (2.10 - 2.03)
Space group	P 6 2 2	P 6 2 2
Unit cell dimensions (Å)	156.42, 156.42, 85.863	156.80, 156.80, 85.60
Unit cell angles (°)	90, 90, 120	90, 90, 120
Total reflections	752,154 (39,936)	1,381,818 (105,151)
Unique reflections	24,683 (1965)	40,437 (3563)
Multiplicity	30.5 (16.9)	34.2 (26.5)
Completeness	0.98 (0.81)	0.97 (0.90)
Mean I/sigma(I)	16.18 (4.53)	25.73 (3.72)
Wilson B-factor	31.09	24
R-merge	0.1953 (0.595)	0.202 (1.548)
R-meas	0.1987 (0.6167)	0.205 (1.581)
R-pim	0.03545 (0.1536)	0.036 (0.315)
CC1/2	0.998 (0.616)	0.997 (0.778)
CC*	0.999 (0.873)	0.999 (0.936)
Reflections used in refinement	24,232 (1965)	39,361 (3563)
Reflections used for R-free	2426 (197)	3891 (350)
R-work	0.1938 (0.2439)	0.202 (0.271)
R-free	0.2299 (0.2806)	0.237 (0.302)
CC(work)	0.938 (0.797)	0.946 (0.831)
CC(free)	0.924 (0.736)	0.936 (0.716)
Number of non-hydrogen atoms	3327	3535
macromolecules	3127	3127
ligands	18	6
solvent	182	402
Protein residues	382	382
RMS (bonds)	0.002	0.003
RMS (angles)	0.5	0.64
Ramachandran favored (%)	98.4	98.66
Ramachandran allowed (%)	1.6	1.34
Ramachandran outliers (%)	0	0
Rotamer outliers (%)	1.51	1.82
Clashscore	1.8	2.63
Average B-factor (Å^2)	42.53	39.38
macromolecules	42.72	38.95
ligands	48.15	36.72
solvent	38.74	42.72
Number of TLS groups	4	4

*Numbers in parentheses are for the highest resolution shell.

Supplementary Table 2. Largest contiguous cavities between peptides and MHC binding grooves in structures of complexes in the HLA-A3 superfamily.

PDB ID	HLA	Peptide length	Volume (Å^3)
2xpg	A3	9	293
3rl1	A3	9	139
3rl2	A3	10	94
6o9b	A3	9	266
6o9c	A3	9	343
7l1b (PI3Kα WT)	A3	9	270
7l1c (PI3Kα neo)	A3	9	401
7mle	A3	9	172
7uc5	A3	9	31
8dvg	A3	10	14
8vcl (PI3Kα neo replicate)	A3	9	284
1q94	A11	9	134
1qvo	A11	10	270
1x7q	A11	9	173
2hn7	A11	10	81
4mj5	A11	11	99
4mj6	A11	11	180
5grd	A11	10	80
5gsd	A11	7	267
5wjl	A11	10	53
5wjn	A11	10	342
6joz	A11	9	282
7m8t	A11	9	223
7ow3	A11	10	59
7s8q	A11	10	340
7s8r	A11	9	102
7s8s	A11	10	61
7wkj	A11	9	96
8rbu	A11	10	113
8rh6	A11	10	102
8rhq	A11	10	27
8i5e	A11	9	49
6j1w	A30	9	148
6j1v	A30	9	436
6j29	A30	9	165
6j2a	A30	9	289
4hwz	A68	9	137
4hx1	A68	9	115
4i48	A68	9	151
6ei2	A68	10	70

Average: 174 Å^3
Standard deviation: 112 Å^3

Supplementary Table 3. Table of antibodies used and their dilutions.

Antibody	Clone	Company	Catalog Number	Dilution
αCD3 APC-H7	SK7	BD	641397	1:20
αCD8 eFluor 450	SK1	Invitrogen	48-0087-42	1:40
αTCR β PerCpCy5.5	H57-597	Invitrogen	45-5961-82	1:40
αCD107a BV650	H4A3	Biolegend	328638	1:40
αTNFα PE	Mab11	Invitrogen	12-7349-82	1:40

Supplementary Table 4. System setups for molecular dynamics simulations.

Traditional and Weighted Ensemble Molecular Dynamics Simulations

TCR-free Systems	***Dimensions (cubic)***	***No. Atoms***	***Waters (R >= 10 Å)***	***Salt***
Neo	100.4 100.1 100.7	86232	26717	12 Na+ (charge neutralized)
WT	101.6 101.4 101.3	89296	27739	12 Na+ (charge neutralized)
Neo - His3+	99.9 100.1 99.9	84528	26149	11 Na+ (charge neutralized)

TCR-bound Systems	***Dimensions (cubic)***	***No. Atoms***	***Waters (R >= 10 Å)***	***Salt***
Neo	164.3 164.3 163.3	397954	128424	23 Na+ (charge neutralized)
WT	162.8 163.0 162.8	389104	125422	18 Na+ (charge neutralized)

Steered Molecular Dynamics Simulations

System	***Dimensions (dodecahedral)***	***No. Atoms***	***Waters***	***Salt***
Neo	9.41630 9.41630 6.65833 0.00000 0.00000 0.00000 0.00000 4.70815 4.70815	60528	18111	0.15 M
WT	9.50390 9.50390 6.72027 0.00000 0.00000 0.00000 0.00000 4.75195 4.75195	62077	18628	0.15 M

Supplementary Note 1. MD simulation checklist

**Reliability and reproducibility checklist for molecular dynamics simulations** ***All boxes must be marked YES by acceptance unless "Response not needed if No".**		**Yes**	**No**	**Response (Please state where this information can be found in the text)**
**1. Convergence of simulations and analysis**				
1a. Is an evaluation presented in the text to show that the property being measured has equilibrated in the simulations (*e.g.* time-course analysis)?		☒	☐	Supp Fig 12
1b. Then, is it described in the text how simulations are split into equilibration and production runs and how much data were analyzed from production runs?		☒	☐	Methods section
1c. Are there at least 3 simulations per simulation condition with statistical analysis?		☒	☐	Results, Methods, Supp Figs. 2 and 10
1d. Is evidence provided in the text that the simulation results presented are independent of initial configuration?		☒	☐	Results, Methods, Supp Figs. 2 and 10
**2. Connection to experiments**				
2a. Are calculations provided that can connect to experiments (*e.g.* loss or gain in function from mutagenesis, binding assays, NMR chemical shifts, J-couplings, SAXS curves, interaction distances or FRET distances, structure factors, diffusion coefficients, bulk modulus and other mechanical properties, *etc*.)?		☒	☐	Simulation results connect to structural, binding, mutation, kinetic, and NMR data
**3. Method choice**				
3a. Do simulations contain membranes, membrane proteins, intrinsically disordered proteins, glycans, nucleic acids, polymers, or cryptic ligand binding?		☐	☒	Response not needed if **No**
3b. Is it described in the text whether the accuracy of the chosen model(s) is sufficient to address the question(s) under investigation (e.g. all-atom vs. coarse-grained models, fixed charge vs. polarizable force fields, implicit vs. explicit solvent or membrane, force field and water model, etc.)?		☒	☐	Implied by convergence of experiment and simulation (also no coarse graining, etc.)
3c. Is the timescale of the event(s) under investigation beyond the brute-force MD simulation timescale in this study that enhanced sampling methods are needed?		☒	☐	
	If **YES**, are the parameters and convergence criteria for the enhanced sampling method clearly stated?	☒	☐	WEMD and SMD used, Fig 6, Supp Figs 6 and 8 and associated text
	If **NO,** is the evidence provided in the text?	☐	☐	
**4. Code and reproducibility**				
4a. Is a table provided describing the system setup that includes simulation box dimensions, total number of atoms, total number of water molecules, salt concentration, lipid composition (number of molecules and type)?		☒	☐	Supp Table 4
4b. Is it described in the text what simulation and analysis software and which versions are used?		☒	☐	Methods
4c. Are other parameters for the system setup described in the text, such as protonation state, type of structural restraints if applied, nonbonded cutoff, thermostat and barostat, etc.?		☒	☐	Methods
4d. Are initial coordinate and simulation input files and a coordinate file of the final output provided as supplementary files or in a public repository?		☒	☐	Yes
4e. Is there custom code or custom force field parameters?		☐	☒	Response not needed if **No**
	If **YES**, are they provided as supplementary files or in a public repository?	☐	☐	
