# Supplementary material for: Dynamic allostery in the peptide/MHC complex enables TCR neoantigen selectivity
Source: Nat Commun. 2025 Jan 20;16:849. doi: 10.1038/s41467-025-56004-8 (PMC11756396; doi:10.1038/s41467-025-56004-8)
Supplement: Supplementary file 2 — Description of Additional Supplementary Files [file 41467_2025_56004_MOESM2_ESM.pdf]

Description of Additional Supplementary Files

Supplementary Movie 1: Trajectory of an under-peptide neoantigen flip from the reverse WEMD simulations. The trajectory begins with pTrp6 of the neoantigen in the TCR-bound neoantigen (the flipped conformation). It flips via the peptidelimbo mechanism to the TCR-free conformation with a final RMSD to the target state of 0.6 Å. The transition occurs at approximately 4 seconds, or approximately 2300 frames into the 5410 frame simulation.
