# Supplementary material for: Dynamic allostery in the peptide/MHC complex enables TCR neoantigen selectivity
Source: Nat Commun. 2025 Jan 20;16:849. doi: 10.1038/s41467-025-56004-8 (PMC11756396; doi:10.1038/s41467-025-56004-8)
Supplement: Supplementary file 4 — Reporting Summary [file 41467_2025_56004_MOESM4_ESM.pdf]

nature portfolio

Corresponding author(s): Brian Baker

Last updated by author(s): Dec 12, 2024

Reporting Summary

Nature Portfolio wishes to improve the reproducibility of the work that we publish. This form provides structure for consistency and transparency in reporting. For further information on Nature Portfolio policies, see our Editorial Policies and the Editorial Policy Checklist.

Statistics

For all statistical analyses, confirm that the following items are present in the figure legend, table legend, main text, or Methods section.

n/a	Confirmed	
☐	☒	The exact sample size (*n*) for each experimental group/condition, given as a discrete number and unit of measurement
☐	☒	A statement on whether measurements were taken from distinct samples or whether the same sample was measured repeatedly
☐	☒	The statistical test(s) used AND whether they are one- or two-sided<br>*Only common tests should be described solely by name; describe more complex techniques in the Methods section.*
☒	☐	A description of all covariates tested
☒	☐	A description of any assumptions or corrections, such as tests of normality and adjustment for multiple comparisons
☐	☒	A full description of the statistical parameters including central tendency (e.g. means) or other basic estimates (e.g. regression coefficient) AND variation (e.g. standard deviation) or associated estimates of uncertainty (e.g. confidence intervals)
☐	☒	For null hypothesis testing, the test statistic (e.g. *F*, *t*, *r*) with confidence intervals, effect sizes, degrees of freedom and *P* value noted<br>*Give P values as exact values whenever suitable.*
☒	☐	For Bayesian analysis, information on the choice of priors and Markov chain Monte Carlo settings
☒	☐	For hierarchical and complex designs, identification of the appropriate level for tests and full reporting of outcomes
☒	☐	Estimates of effect sizes (e.g. Cohen's *d*, Pearson's *r*), indicating how they were calculated

Our web collection on statistics for biologists contains articles on many of the points above.

Software and code

Policy information about availability of computer code

Data collection	Biacore control software 2.01 (SPR), NanoTemper PR.ThermControl v2.1 (thermal stability), Amber18 (MD), Topspin 4 (NMR), Amber18 (MD), H++ 4.0 (pKa), WESTPA 1.0 (MD), GROMACS 2022 (MD), BD FACSDiva v9 (flow)
Data analysis	Biacore T200 evaluation software v3.0 (SPR processing), Biaevaluation 4.1 (SPR fitting), OriginPro v2024 (binding, thermal stability), CAVER Analyst 2.0 (cavity analysis), MolVol 1.1 (visualization), Discovery Studio 2023 (visualization and OMIT maps), MATLAB R2022a (MD analysis), Mestrelab MNova NMR 15 (NMR), PyMOL 3 (visualization), UCSF Chimera 1.16 (visualization), HKL2000 v721 (X-ray), Phaser v2.8.1 (X-ray), PHENIX v1.13-2998 (X-ray), Coot 0.8.9 (X-ray), CCP4/Aimless 2023 (X-ray), CCPTRAJ 18 (MD), FlowJo version 10.6.2 (flow)

For manuscripts utilizing custom algorithms or software that are central to the research but not yet described in published literature, software must be made available to editors and reviewers. We strongly encourage code deposition in a community repository (e.g. GitHub). See the Nature Portfolio guidelines for submitting code & software for further information.

Data

Policy information about availability of data

All manuscripts must include a data availability statement. This statement should provide the following information, where applicable:

- Accession codes, unique identifiers, or web links for publicly available datasets
- A description of any restrictions on data availability
- For clinical datasets or third party data, please ensure that the statement adheres to our policy

Data are available upon request from the authors or from the Zenodo repository under accession code 14392129 . Crystallographic data are available from the PDB with accession codes 8VCL (neoantigen/HLA-A3) and 9ASG (Bta-substituted neoantigen/HLA-A3). NMR data are under the Zenodo link above and also at the BMRbig database under accession code BMRbig105. Links to all databases and accession codes are in the manuscript.

Research involving human participants, their data, or biological material

Policy information about studies with human participants or human data. See also policy information about sex, gender (identity/presentation), and sexual orientation and race, ethnicity and racism.

Reporting on sex and gender	n/a
Reporting on race, ethnicity, or other socially relevant groupings	n/a
Population characteristics	n/a
Recruitment	n/a
Ethics oversight	n/a

Note that full information on the approval of the study protocol must also be provided in the manuscript.

Field-specific reporting

Please select the one below that is the best fit for your research. If you are not sure, read the appropriate sections before making your selection.

☒ Life sciences ☐ Behavioural & social sciences ☐ Ecological, evolutionary & environmental sciences

For a reference copy of the document with all sections, see nature.com/documents/nr-reporting-summary-flat.pdf

Life sciences study design

All studies must disclose on these points even when the disclosure is negative.

Sample size	Sample sizes were fixed based on the experiments described: neoantigen/HLA-A3 vs. WT/HLA-A3, or in two cases including modified peptides (Bta of fluorine), with two TCRs (TCR3 and TCR4). Beyond these there were no additional experimental samples other than positive and negative peptide/MHC and scTv controls. Concentrations chosen for titrations were to ensure saturation, or in the case of negative controls, to ensure high values that accurately reported a lack of binding or functional responses.
Data exclusions	No data were excluded.
Replication	Experiments and molecular dynamics simulations were replicated as described in the text: simulations 3-4 times, SPR 3-6 times with duplicate injections, and functional titrations 3 times.
Randomization	As a structural and biophysical study using recombinant protein or simulations, with in vitro functional data for confirming solution measurements, blinding was not performed. In titration experiments, dilutions were made with robotic assistance in a controlled fashion in order of increasing concentration, obviating randomization. Crystal screening was performed similarly by robotics, obviating randomization.
Blinding	As a structural and biophysical study using recombinant protein or simulations, with in vitro functional data for confirming solution measurements, blinding was not performed. or example, in titration experiments, dilutions were made with robotic assistance in a controlled fashion in order of increasing concentration, obviating blinding. Crystal screening was performed similarly by robotics with a need to link conditions to crystals, obviating blinding.

Reporting for specific materials, systems and methods

We require information from authors about some types of materials, experimental systems and methods used in many studies. Here, indicate whether each material, system or method listed is relevant to your study. If you are not sure if a list item applies to your research, read the appropriate section before selecting a response.

Materials & experimental systems

n/a	Involved in the study
☐	☒ Antibodies
☐	☒ Eukaryotic cell lines
☒	☐ Palaeontology and archaeology
☒	☐ Animals and other organisms
☒	☐ Clinical data
☒	☐ Dual use research of concern
☒	☐ Plants

Methods

n/a	Involved in the study
☒	☐ ChIP-seq
☐	☒ Flow cytometry
☒	☐ MRI-based neuroimaging

Antibodies

Antibodies used: anti-CD3-APC-H7 (Clone SK7, BD cat. no. 641397, lot no. 3321603), anti-CD8-efluor450 (Clone SK1, Invitrogen cat. no. 48-0087-42, lot no. 2466961), anti-mouse TCR-PerCpCy5.5 (Clone H57-597, Invitrogen cat. no. 45-5961-82, lot no. 2804610), anti-CD107a (Clone H4A3, Biolegend cat. no. 328638, lot no. B407829) and anti-TNFα-PE (Clone Mab11, Invitrogen cat. no. 12-7349-82, lot no. 2622555).

Validation: All antibodies used are common and have been characterized and documented in the literature. Validation reports linked to lot numbers are available from the manufacturers using the websites https://regdocs.bd.com/regdocs/qcinfo, https://www.thermofisher.com/us/en/home/support/documents-certificates.html, and https://www.biolegend.com/en-gb/certificate-of-analysis

Eukaryotic cell lines

Policy information about cell lines and Sex and Gender in Research

Cell line source(s): The retroviral packaging line 293-GP was purchased from Takara Bio (cat #631530). COS-7 cells were obtained through an MTA from S. A. Rosenberg (NCI, Bethesda, USA). Primary T cells for TCR transduction were isolated from deidentified healthy-donor human peripheral blood leukopaks purchased from StemCell Technologies (Catalog 200-0092).

Authentication: Cell lines are well characterized. 293-GP are authenticated by manufacturer. Further authentication was not performed.

Mycoplasma contamination: All cell lines are routinely tested in-house for mycoplasma and were confirmed to be negative at the time of use.

Commonly misidentified lines (See ICLAC register): n/a

Plants

Seed stocks: n/a

Novel plant genotypes: n/a

Authentication: n/a

Flow Cytometry

Plots

Confirm that:

☒ The axis labels state the marker and fluorochrome used (e.g. CD4-FITC).

☒ The axis scales are clearly visible. Include numbers along axes only for bottom left plot of group (a 'group' is an analysis of identical markers).

☒ All plots are contour plots with outliers or pseudocolor plots.

☒ A numerical value for number of cells or percentage (with statistics) is provided.

Methodology

Sample preparation

TCR-transduced T cells were stimulated by peptide-pulsed tumor targets in the presence of anti-CD107A- BV650 and Golgi Block. After 6h, cells were washed in 1x PBS and surface labeled with Live/Dead fixable dye, anti-CD3-APC-H7, anti-CD8-efluor450 and anti-mouse TCR- PerCpCy5.5 for 30 min at 4C. Cells were washed with 1x PBS and then fixed and permeabilized for 15’ at 4C. Surface-labeled cells were then washed with 1x perm-wash buffer and labeled with anti-TNFα-PE for 30 minutes at 4°C in perm-wash buffer. Cells were washed with perm-wash buffer and suspended in 2% FBS in PBS prior to acquisition.

Instrument

BD LSRFortessa X20 flow cytometer.

Software

Acquisition was done using BD FACSDiva v9 and analysis was performed using FlowJo software version 10.6.2.

Cell population abundance

CD8+ T cells were enriched prior to TCR-transduction. TCR-T cells are represented at >75%.

Gating strategy

Live T cells were gated using FSC vs SSC, singlet and viability gating. Downstream gating was determined using the T cell lineage marker expression, CD3 and CD8. CD3+CD8+ cells are gated on transduced TCR expression to identify the population of interest. Gating for T cell functional markers (CD107a or TNFα) were assigned based on biological controls.

☒ Tick this box to confirm that a figure exemplifying the gating strategy is provided in the Supplementary Information.
